# PI3K Signaling Pathway Inhibitor Affects Myeloma Cells in a Culture-Dependent Manner

**DOI:** 10.34172/apb.025.42774

**Published:** 2025-04-24

**Authors:** Mehrnaz Janfada, Sadaf Vahdat, Saeid Kaviani

**Affiliations:** ^1^Department of Hematology, Faculty of Medical Sciences, Tarbiat Modares University, Tehran, Iran; ^2^Applied Cell Sciences Division, Department of Hematology, Faculty of Medical Sciences, Tarbiat Modares University, Tehran, Iran

**Keywords:** Multiple myeloma, Tumor-associated macrophages, 3D cell culture, PI3K inhibitor

## Abstract

**Purpose:**

The survival and progression of multiple myeloma (MM) cells rely heavily on supportive factors and cells within the MM microenvironment, notably macrophages. The PI3K signaling pathway plays a crucial role in both myeloma cells survival and macrophage polarity, making it a potential target for altering the MM microenvironment dynamics.

**Methods:**

In this study, the impact of LY294002, a PI3K signaling pathway inhibitor, on the viability of U266 myeloma cells in mono-culture and MM patient-derived bone marrow mononuclear cells (BM-MNCs) in co-culture was investigated. Additionally, the effect of treatments on the M1/M2 macrophage ratio was assessed. Cultures were conducted in both two-dimensional (2D) matrix-free and fibrin gel-based three-dimensional (3D) environments.

**Results:**

The treatment significantly increased U266 cell death in 2D cultures, dose-dependently compared to control. However, this effect was not replicated in 3D cultures. In both 2D and 3D cultures, the percentages of cells in G0/G1 phase were dose-dependently increased, compared to the untreated control. However, the percentages of cells in S and G2/M phases in both 2D and 3D cultures were dose-dependently decreased, compared to control. Treatment of BM-MNCs with LY294002 showed patient- and culture-dependent patterns of CD138^+^ myeloma cell death and M1/M2 macrophage ratio, contrasting the observed consistent responses in U266 mono-culture.

**Conclusion:**

LY294002 affected U266 cell viability and cell cycle in a dose-dependent manner in 2D mono-cultures. However, its impact varied in 3D cultures. Treatment of MNCs showed varied responses based on individuals and culture conditions, underscoring the need for more similar tumor microenvironment (TME) recapitulation for drug screening.

## Introduction

 Multiple myeloma (MM) is a clonal plasma cell malignancy and the second most common hematologic cancer after non-Hodgkin’s lymphoma.^1,2^ The exact cause of MM is not well understood.^2,3^ Despite the made efforts, this cancer is still incurable and needs novel therapeutic strategies by targeting the tumor microenvironment (TME)^4,5^; it has been confirmed that the TME plays a crucial role in the development and progression of the MM disease.^6,7^ Malignant cells interact with and influence the surrounding cells, leading to the generation of a supportive environment in favor of their growth.^8^ Moreover, myeloma cells can evade the immune system by altering the function of immune cells in the microenvironment.^9-12^ Macrophages are abundant cells with high flexibility in the MM microenvironment, and tumor-associated macrophages (TAMs) represent significant impacts on the progression of MM disease rather than contribution to anti-cancer immune responses, through secretion of growth factors that suppress the immune system responses against myeloma cells, support the growth and proliferation of MM cells and promote angiogenesis.^13-15^ Drug resistance can also be resulted from the interactions of myeloma cells with macrophages.^14,16^

 Based on the environmental factors, TAMs polarized to two distinct subtypes, M1 and M2 phenotypes.^14^ The classically activated macrophages (M1) with anti-tumor activity and pro-inflammatory properties release inflammatory cytokines. The alternatively activated macrophages (M2) have anti-inflammatory and immunosuppression properties which can promote angiogenesis and tumor progression.^14,17^ Therefore, targeting macrophages and changing the balance of M1 and M2 phenotypes would be potential effective therapeutic approaches in this field.^14,18,19^

 The phosphatidylinositol 3-kinase (PI3K) signaling pathway is considered as a central regulator of macrophage polarization process.^20-24^ It has been accepted that this signaling pathway involves in many processes in the MM disease and can be activated by many secreted cytokines in the MM microenvironment, including IL-6, IGF-1, VEGF and CXCL12.^25^ Accordingly, several studies have exhibited the potential of PI3K signaling pathway manipulation to control either the survival and proliferation of MM cells or the polarization of macrophages.^21,26-29^

 In various studies, the effect of small molecular PI3K inhibitors, such as TG100-115, BEZ235, SRX3207 and SF2523, on macrophage reprogramming has been assessed in two-dimensional (2D) culture conditions.^30-33^ On the other hand, several studies have shown that the inhibition of PI3K signaling pathway can inhibit myeloma cell growth and proliferation in 2D cultures.^27,34-36^ However, the effect of treatment with inhibitors of this signaling pathway on both myeloma cells and macrophages is required to be investigated in a comprehensive study. Since the interaction of myeloma cells with other TME components plays an important role in the activation of PI3K signaling pathway as well as the regulation of cell responses to the drug, more similar recapitulation of TME interactions is highly demanded for precise drug screenings.^37,38^ In this regard, 2D mono-cultures cannot simulate the complexity of the bone marrow (BM) microenvironment, while 3D co-cultures can represent the physiological conditions of TME in a more similar way.^39,40^ The superiority of 3D cultures over 2D ones has been exhibited in distinct aspects, including the cytokine secretion and drug resistance of MM cells.^41-45^ Accordingly, we hypothesized that the efficacy of the PI3K signaling pathway inhibitor in reducing myeloma cell viability would be influenced by culture conditions and the presence of other cellular components within the myeloma microenvironment. Therefore, to comprehensively assess these effects on the effectiveness of the treatment, we evaluated the impact of LY294002, a broad-spectrum inhibitor of PI3K signaling pathway,^46^ on myeloma cell death under both 2D and for the first time, plasma-derived fibrin gel-based 3D culture conditions. Moreover, we examined whether mono-culture (U266 MM cells alone) versus co-culture (MM cells within patient BM-derived mononuclear cells (MNCs)) influenced myeloma cell survival following treatment. Finally, we assessed how LY294002 modulated the M1/M2 macrophage ratio in both 2D and 3D culture conditions. This study would provide novel insights into the role of the microenvironment in modulating therapeutic responses and highlighted the importance of 3D patient-derived models in evaluating drug efficacy.

## Materials and Methods

###  Study design

**Figure 1:**
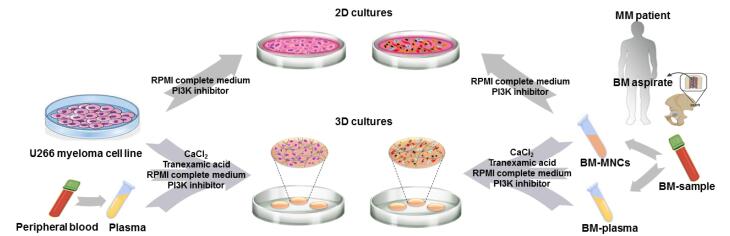
The schematic representation of our study design.The effect of treatment with the PI3K signaling pathway inhibitor was evaluated on the myeloma cell death in two culture conditions: 2D and fibrin gel-based 3D cultures. Moreover, in order to assess the impact of mono-culture of MM cells versus co-culture of MM microenvironment cells on the myeloma cell death after treatment, both U266 myeloma cell line and mononuclear cells (MNCs) derived from BM samples of MM patients were used. PB- and BM-derived plasma samples were used for the generation of fibrin gels for 3D culture of U266 cells and MNCs, respectively. MM; multiple myeloma, BM; bone marrow, MNCs; mononuclear cells, 2D culture; two-dimensional culture, 3D culture; three-dimensional culture

 As presented in Figure 1, in this study, it was tried to evaluate the effect of treatment with PI3K signaling pathway inhibitor, LY294002, on the survival of myeloma cells and the polarization of macrophages in two different culture conditions: 2D matrix-free and fibrin gel-based 3D cultures. Assessments were carried out on both U266 myeloma cell line and MM patients-derived MNCs. Two different concentrations of the inhibitor (10 and 25 μM) were used and assessments were performed at days one, two and three after treatments. Cultures without treatments were considered as controls.

###  2D culture of U266 cell line 

 U266 MM cells were cultured in the complete culture medium, containing RPMI-1640 medium (Cat. No.: 035-51800, Gibco, USA) supplemented by 1% L-glutamine, 10% fetal bovine serum (FBS; Cat. No.: BI-1201, Bioidea, Iran) and 1% penicillin/streptomycin (Cat No.: BI-1203, Bioidea, Iran), at 37 °C, 5% CO_2_ and 95% humidity.^47^ Culture medium was refreshed every three days and cells were sub-cultured at 5 × 10^5^ cells/ml seeding density.

###  Isolation of MM patients-derived plasma and MNCs 

 Primary MNCs and plasma fractions were isolated from BM samples of five MM patients. All samples were collected after obtaining written informed consent from the patients. The demographic patients’ data is presented in Table 1. Plasma fractions were obtained by the centrifugation of BM samples at 1500 rpm for 10 minutes at 22 °C. Plasma samples derived from different patients were pooled in order to remove inter-individual variables. Thereafter, Ficoll-Paque (Cat. NO.: BD0018, DNA Biotech, Iran) was used to isolate MNCs by density gradient centrifugation.^48^

**Table 1:** The demographic data of patients and the characteristics of their samples

**Characteristic **	**Value **
No. of patients	5
Age (y) (Mean ± SD)	65 ± 4.3
Gender	Male
Race	Iranian
Disease stage (No. of patients)	Newly diagnosed: 4 Relapse/Progression: 1
Sample volume	2-3 ml
Isolated MNCs/mL ( × 10^6^; Mean ± SD)	4.0 ± 0.5
CD138^+^ myeloma cells (%; Mean ± SD)	15.9 % ± 6.0 %
CD68 ^+^ macrophage cells (%; Mean ± SD)	32.4 % ± 4.5 %

###  Isolation of plasma from peripheral blood (PB) samples

 Plasma samples were isolated from PB of three volunteers. Two ml of blood from each donor collected in EDTA anticoagulant were centrifuged at 1500 rpm for 10 minutes at 22 °C to separate the plasma fraction. To remove donor-specific factors that could impact the fibrin gel generation, plasma samples were pooled.^47^

###  Generation of fibrin gels 

 For 3D culture of cells, fibrin gels were generated as previously described.^47^ Briefly, PB- and BM-derived plasma samples were mixed with 1 mg/mL calcium chloride (Cat. No.: C7902, Sigma-Aldrich, Germany) and 5 mg/ml tranexamic acid (Caspian Tamin Pharma Co., Iran) to be used for gel-based culture of U266 cells and patient-derived MNCs, respectively. U266 cells or MNCs were added to the plasma mixture before gelation and the volume was increased with the complete culture medium to reach the density of 1 × 10^5^ cells/100 µL,^49^ in the presence or absence of the LY294002 hydrochloride (Cat. NO.: 1130, Tocris Bioscience, UK). The cell/gel mixture was incubated at 37 °C, 5% CO_2_ and 95% humidity. After two hours, the complete culture medium was added on top of the formed gels.

###  Flow cytometry assessment

 Flow cytometry of surface markers was performed as previously described.^47^ Briefly, isolation of cells cultured inside gels was performed by 5 mg/ml collagenase type I (Cat. NO.: 17100-017, Gibco, USA) enzymatic digestion. 2D and 3D cultured cells (after isolation) were washed with PBS (Cat. NO.: S0201, BioBench, Iran) and thereafter, 1 × 10^5^ cells were incubated with CD138 (Cat. NO.: IQP-153F, IQ Products, Netherlands), CD68 (Cat. NO.: 333808, BioLegend, USA), CD86 (Cat. NO.: 305406, BioLegend, USA) or CD206 (Cat. NO.: 321106, BioLegend, USA) antibodies, at 4 °C for 45 minutes. CD138 and propidium iodide (PI) (Cat. NO.: P4170, Sigma-Aldrich, Germany) double staining was performed to analyze the survival of MM cells. For cell cycle assessment, U266 cells were fixed in 70% ethanol and after washing with PBS, were stained with PI in the presence of 100 μL of RNase A for 30 minutes. After washing unbound antibodies and dyes, cell analysis was carried out using flow cytometer system (BD FACSCanto II, BD Biosciences, USA). Flow cytometry data was analyzed with Flowing Software 2.4.1.

###  Statistical analysis

 The experiments on U266 cells were performed with at least three independent replications. Statistical analysis was carried out by the ANOVA test (Tukey post hoc) using SPSS software (version 22). The significance of differences between groups was determined using the *P* ≤ 0.05 significance level. The data is displayed as mean ± standard deviation in the graphs.

## Results

###  The effect of PI3K signaling pathway inhibitor on U266 cells

**Figure 2:**
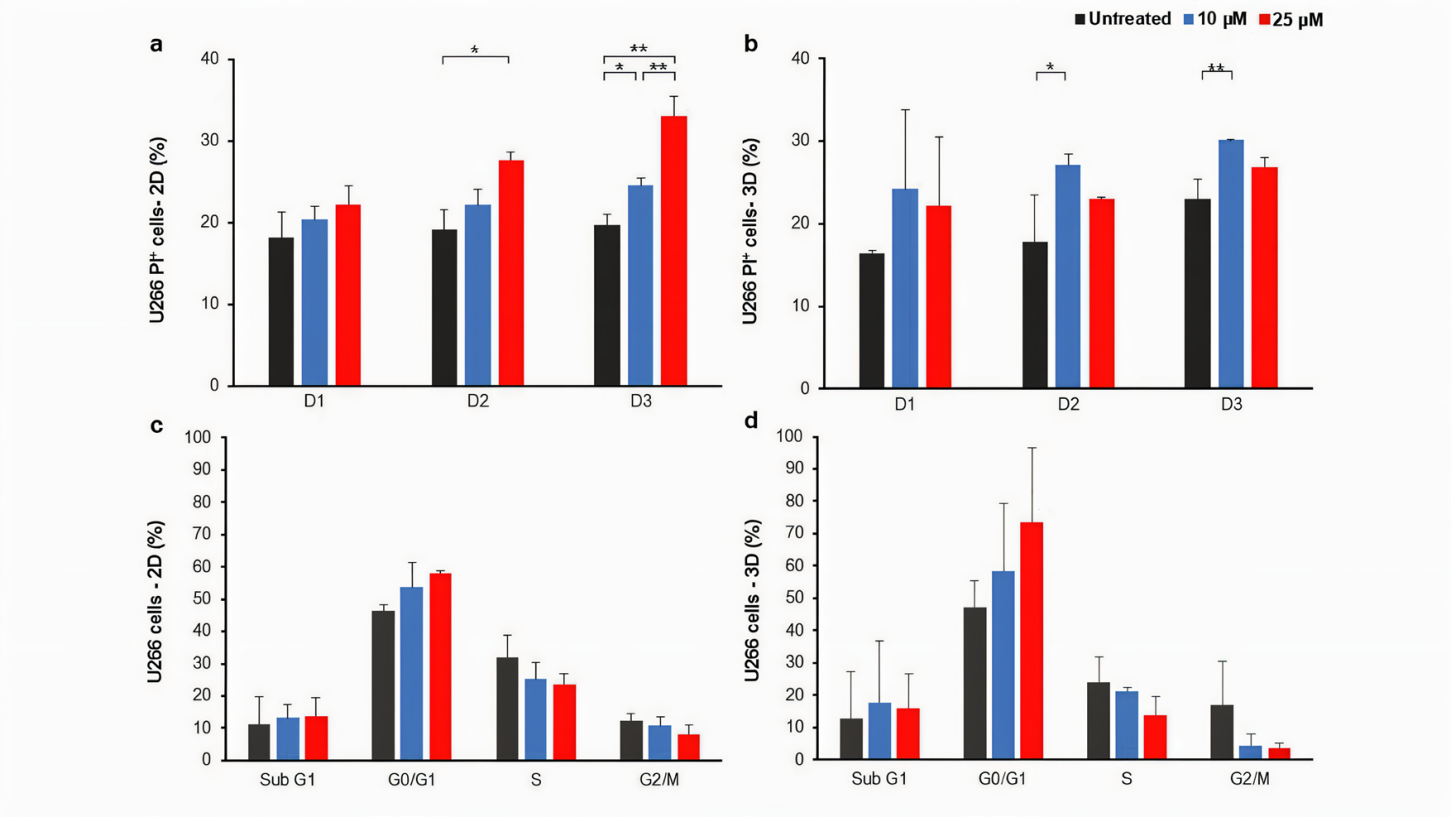
The effect of LY294002 on the U266 cell death and cell cycle in 2D and 3D culture conditions.The effect of LY294002 on the U266 myeloma cells death one, two and three days post-treatment in 2D (a) and 3D (b) culture conditions was not the same. * *P* < 0.05 and ** *P* < 0.01. The percentage of cells in each phase of the cell cycle at day two post-treatment with LY294002 in 2D and 3D culture conditions is presented in (c) and (d), respectively. The mean ± standard deviation is used to present all data. Graphs represent the obtained results from three independent experimental replicates. D; Day, 2D; two-dimensional culture, 3D; three-dimensional culture

 In order to investigate the effect of treatment with LY294002 on the viability/death of myeloma cells, we started with U266 cells, which were treated with two concentrations of LY294002 (10 and 25 μM) in both gel-free 2D and gel-based 3D culture conditions and the effects of treatment on the death and cell cycle of U266 cells were assessed. As shown in Figure 2a, the treatment of U266 cells in the 2D culture condition resulted in the increased cell death in a dose-dependent manner compared to the untreated control group although the differences between some groups were not statistically significant. Moreover, 25 μM of LY294002 could significantly increase the percentage of PI^+^ cells in the 2D culture of U266 cells compared to the untreated group, at days two and three post-treatment. However, the significantly increased percentage of PI^+^ cells was observed when 3D cultured U266 cells were treated with 10 μM of LY294002 compared to those of the untreated group, at days two and three post-treatment (Figure 2b). The cell cycle evaluation of 2D cultured U266 cells at day two post-treatment revealed a dose-dependent increase of cells in the G0/G1 phase compared to the untreated control group (Figure 2c and Figure S1). Moreover, dose-dependent decreases of cells in both S and G2/M phases were observed compared to the untreated control cells (Figure 2c). The dose-dependent pattern of cell distribution in G0/G1, S and G2/M phases were similarly observed in 3D cultured cells after treatments (Figure 2d and Figure S1).

###  The effect of PI3K signaling pathway inhibitor on MM patient-derived primary cells 

 In the next step, in order to make the condition more complex, co-culture of TME cells was performed in both 2D and 3D culture conditions. BM-derived MNCs were isolated from five male MM patients, with the average age of 65 ± 4.3 years (Table 1). Four out of five patients were newly diagnosed and one patient had relapsed disease after four months of taking the last doses of medicine. As presented in the Table 1, 15.9 % ± 6.0 % of isolated MNCs were CD138 positive.

**Figure 3:**
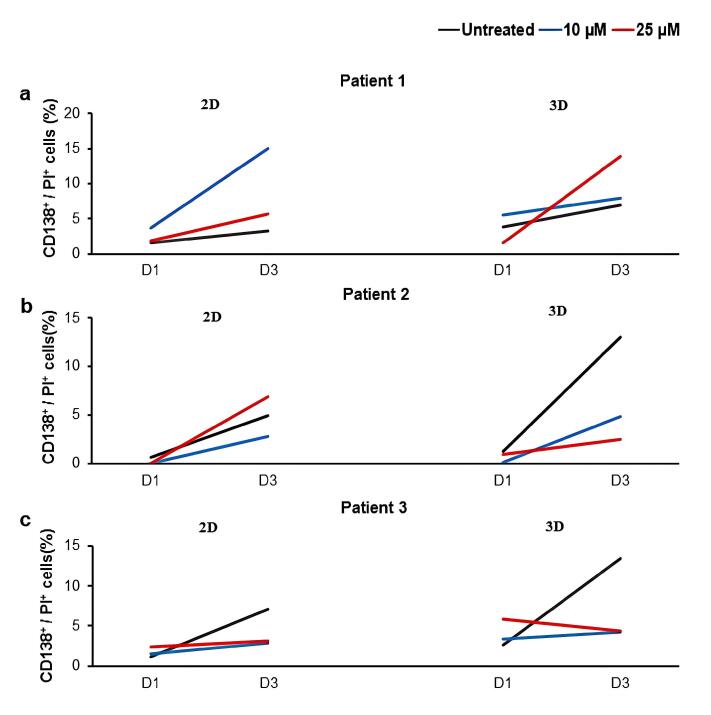
The effect of LY294002 on the death of primary myeloma cells in 2D and 3D culture conditions.The percentage of CD138^+^/PI^+^ cells in MNCs derived from three patients after treatments with two concentrations of LY294002 (10 and 25 μM) in 2D and 3D culture conditions are presented in (a-c). Cultures without the inhibitor (untreated) were considered as controls. D; Day, 2D; two-dimensional culture, 3D; three-dimensional culture

 Similar to U266 cell, isolated MNCs were treated with two concentrations of LY294002 (10 and 25 μM) in both 2D and 3D culture conditions. As presented in Figure 3 and Figure S2, different patient-dependent and culture-dependent responses were observed after LY294002 treatment. For instance, the treatment of patient 1-derived MNCs in the 2D culture condition resulted in an increase in the percentage of CD138^+^/PI^+^ cells with 10 μM LY294002 over three days of treatment. The pattern was different in the 3D culture of patient 1-derived MNCs, which showed increased percentages of dead cells with 25 μM LY294002 compare to 10 μM LY294002 and untreated groups over three days (Figure 3a). In contrast, the increased percentage of CD138^+^/PI^+^ cells in the 2D culture condition was observed when patient 2 sample was treated 25 μM LY294002. However, there was no increase of CD138^+^/PI^+^ cells in treated groups compared to the untreated group in the 3D culture condition (Figure 3b). Similarly, the treatment of patient 3-derived MNCs in both 2D and 3D culture conditions resulted in no increase of CD138^+^/PI^+^ cells over three days in treated groups compared to the untreated group (Figure 3c).

###  The effect of PI3K signaling pathway inhibitor on MM patients’ macrophages ratio

**Figure 4:**
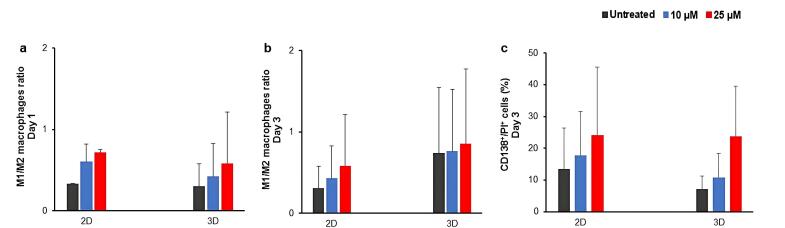
The effect of LY294002 on the ratio of M1/M2 macrophages and the death of primary myeloma cells after three days treatment.The average ratio of M1/M2 macrophages at days one and three post-treatment are presented in a and b, respectively. The average percentage of dead myeloma cells at day three post-treatment is presented in c. Cultures without the inhibitor (untreated) were considered as controls. D; Day, 2D; two-dimensional culture, 3D; three-dimensional culture

 As PI3K signaling pathway is involved in both survival of MM cells and macrophage fate, the effect of treatment with LY294002 was assessed on the ratio of M1/M2 macrophages. Similar to the previous step, the treatment of isolated MNCs with LY294002 resulted into patient-dependent and culture-dependent changes in the percentages of M1 and M2 macrophages (Figures S3, S4 and S5). To evaluate the pattern of macrophage phenotype changes, the ratio of M1/M2 macrophages was measured (Figure 4a and 4b). Accordingly, the measured ratio of M1/M2 macrophages exhibited a dose-dependent increasing pattern in both 2D and 3D culture conditions (Figure 4a and b). Notably, the increasing pattern of the ratio of M1/M2 macrophages was correlated with the increased percentages of CD138^+^/PI^+^ cells in treated groups compared to the untreated groups (Figure 4c and Figure S6).

## Discussion

 It has been demonstrated that the PI3K signaling pathway is an active player in the MM progression, and its inhibition results into the prevention of the proliferation and growth of myeloma cell lines.^27,50^ Activation (phosphorylation) of Akt mainly through the PI3K signaling in myeloma cells is accompanied by the poor prognosis and the reduced patient survival. In fact, the activation of the PI3K/Akt signaling pathway is one of the causes of drug resistance in MM, leading to the prevention of apoptosis and therefore, cancer progression.^51-53^ Targeting this signaling pathway has been used to increase the sensitivity of myeloma cells to anti-cancer treatments.^27,28,52^ On the other hand, the activity of this signaling pathway has also been observed in the macrophage polarization, an important cellular phenomenon occurred in the MM microenvironment.^21,24^ However, most of our knowledge has gained from the 2D mono-culture of tumor cell lines, which cannot appropriately replicate the 3D complex and heterogeneous microenvironment of a cancer.^54^ Therefore, in this study, for the first time it was tried to evaluate the impact of treatment with the inhibitor of PI3K signaling pathway, LY294002, both on the survival of myeloma cells and the ratio of macrophages in 2D and 3D cultures.

 LY294002 small molecule is the first generation of the PI3K pathway inhibitor, which is widely used to decrease tumor growth, inhibit tumor invasion and migration, and sensitize various tumors to chemotherapy.^55-59^ Accordingly, treating U266 MM cells with LY294002 showed low expression levels of BCL-2, Cyclin D1, Cyclin E, PI3K, and AKT in the treated cells, which led to the prevention of U266 proliferation.^60^ While we did not specifically determine the IC50 value of LY294002 in this study, previous reports indicate that 10 μM and 25 μM are within the effective range for inducing apoptosis and inhibiting proliferation in MM cell models^60-62^ and therefore, these two concentrations were used in our study. Since drug efficacy can vary based on culture conditions, cell-cell interactions, and extracellular matrix (ECM) influences, we aimed to assess whether the reported effective doses would retain their impact in our experimental setup. Accordingly, U266 myeloma cell line was utilized as a control to assess whether the reported effects of LY294002 on MM cells could be replicated in our study.^60,61^ In addition, MNCs derived from patients’ BM samples were cultured to partially represent the myeloma TME.^49,63,64^ PB- and BM-derived fibrin gels were generated to culture U266 cells and MNCs, respectively, in more similar conditions to the cancer microenvironment for small molecule testing.^47,49,65,66^ Various studies showed that cells in 3D cultures exhibit the highest level of drug resistance, and accordingly, the results of 3D cultures can fill the gap between 2D cultures and animal models and may be closer to the clinical responses of patients, moving towards achieving personalized treatments for MM patients.^49,65,67-69^

 Our results revealed the time- and LY294002 dose-dependent U266 myeloma cell death after treatment with the small molecule in the 2D culture condition, which was in line with the results of other similar studies.^60,61^ In addition to the effectiveness of inhibitor treatment on the cell death, similar to the Wang et al. study,^60^ we showed that treatment with the inhibitor led to the increased percentage of U266 cells in G0/G1 phase in a dose-dependent manner in 2D cultures. Moreover, the percentages of cells in S and G2/M phases were decreased in a dose-dependent manner, which was consistent with other studies performed in 2D cultures.^27,70^ Different studies have shown that inhibition of PI3K signaling pathway can inhibit myeloma cell growth and proliferation in 2D cultures. For example, it has been shown that treatment with a novel broad-spectrum PI3K inhibitor, Compound A, results into the decreased survival, apoptosis induction, and G1 phase arrest of ARP1, ARK, MM.1S, MM.1R, CAG, and U266 myeloma cell lines as well as primary myeloma cells in dose and time-dependent manners.^36^ In another study, LY294002 treatment inhibited the RPMI 8226 cell migration and VEGF secretion.^71^ Inhibiting the PI3K signaling pathway using BKM120 inhibitor also resulted into the decreased survival of KMM-1 and RPMI 8226 cells through the caspase-3-dependent apoptosis induction. Moreover, G2/M phase arrest was reported in both KMM-1 and RPMI 8226 cell lines, which was through increased expression of SIRT1. However, it was also shown that the percentages of cells in G1 and S phases of the cell cycle were decreased. Moreover, the data suggests that different cell lines showed different sensitivities to inhibitors; RPMI 8226 showed less sensitivity to the inhibitor due to the expression of the wild-type phosphatase PTEN.^27^ BENC-511 is a potent compound for inactivation of PI3K and its downstream signaling, AKT, mTOR, p70S6K, and 4E-BP1. BENC-511 treatment increased the apoptosis of myeloma cell lines, such as RPMI 8226 and U266, by caspase 3 and PARP cleavage. However, the inhibitory effect was decreased with the addition of IL-6 and IGF-1, two primary compounds for inducing the PI3K signaling pathway in myeloma cells. Moreover, the usage of BENC-511 inhibitor could successfully reduce the tumor growth in mouse xenograft models over a period of three weeks.^50^ The selective PI3K-α inhibitor, BYL719, also reduced the survival and proliferation of OPM1/2, RPMI 8226, U266, MM1s, NCI-H929 myeloma cell lines, as well as CD138^+^ primary cells isolated from BM samples of three MM patients. Similar to our results, cell cycle analysis showed that the treatment with the inhibitor led to an increase in the percentage of MM1S cells in the G1 phase and a decrease in the percentage of MM1S cells in the S phase with a dose-dependent pattern.^70^ On the other hand, due to the critical role of PI3K signaling pathway, combinatory therapies using different inhibitors have been used in various studies to increase the cell sensitivity to drugs. For instance, the combination of LY294002 and different compounds such as PIM kinase inhibitors, homoharringtonine, and rapamycin, showed synergistic inhibitory effects on the survival of RPMI 8226 and U266 myeloma cell lines.^61,72,73^

 On days two and three after U266 cells treatment in the 2D culture, the percentage of dead cells was significantly higher in the 25 μM treated group compared to other groups. However, in the 3D culture condition, 10 μM of the inhibitor had a significantly greater effect on the U266 cell death compared to the untreated control group. Similarly, in another study, it has been shown that different concentrations of chemicals have distinct effects on myeloma cells in 2D and 3D cultures.^49^ In addition, the patterns of cell distribution in different cell cycle phases were different between 2D and 3D cultured cells; the observed different pattern of cell behavior in 2D and 3D cultures was in line with other studies.^43,44,49,74-77^ It should be noted that we performed cell cycle analysis at day two post-treatment, as this time point may represent a critical window for assessing the direct effects of PI3K signaling pathway inhibitor on cell cycle progression. Previous research has shown that PI3K inhibitors primarily exert their effects on cell cycle checkpoints at 48 hours of treatment, influencing cell cycle arrest before other downstream mechanisms, such as apoptosis or adaptive resistance, become dominant.^78,79^ At later time points, apoptotic clearance of affected cells may obscure the primary cell cycle alterations induced by the treatment. Future studies may further explore time-dependent variations in cell cycle dynamics by incorporating additional time points.

 Treatment of cultured MNCs in 2D and 3D culture conditions with LY294002 resulted into the patient-dependent patterns of cell death, different from those of U266 cells. In this regard, it has been demonstrated that myeloma cells have complicated and dynamic interactions with other microenvironment cells, both affecting them and being affected by them.^49,74^ Consistently, it has been shown that the co-culture of myeloma cell lines, U266 and RPMI 8226, and primary CD138^+^ myeloma cells with human stromal HS-5 cells reduced the death of MM cells after drug treatment and also increased the drug resistance.^61^ Besides to the probable effect of cell-cell interactions on the survival of myeloma cells in treated MNC groups compared to the U266 groups, the source of plasma for fibrin gel generation in 3D cultures might be another factor affecting the different pattern of cell death between MNCs and U266 cells. Plasma fractions were derived from patients’ BM samples for the 3D culture of MNCs; however, as we aimed to compare the patient-dependent disease models with the available and reproducible cell line-dependent models, PB-derived plasma samples were used for the 3D culture of U266 cells. Patient-derived plasma samples are enriched for myeloma supportive compounds.^80^ Moreover, it is also shown that the cytokine and growth factor contents of plasma samples derived from BM and PB of patients are different.^80,81^

 Considering the culture condition, in 2D cultures, after three days of treatment with LY294002, a concentration of 10 μM led to myeloma cell death in one patient, whereas 25 μM was required to induce a comparable effect in another patient, compared to the control group. However, in the third patient, none of the tested concentrations resulted in increased cell death compared to the control group. In contrast, in 3D cultures, a concentration of 25 μM led to increased cell death in one patient, while in two other patients, neither 10 μM nor 25 μM of LY294002 had a significant impact on myeloma cell death compared to the control. These findings suggest that the pattern of myeloma cell death differs significantly between 2D and 3D culture systems, likely due to differences in cell-cell and cell-matrix interactions, drug penetration, and microenvironmental influences. Consistently, some other studies exhibited the distinct patterns of cell responses to treatments in 2D and 3D cultures; in 3D environments, myeloma cells exhibit a more physiologically relevant morphology, altering intracellular signaling pathways, which may contribute to reduced sensitivity to PI3K inhibition.^49,74^ The usage of fibrin gels for the 3D culture of MM cells might be another factor affecting the obtained data, as fibrin gels could affect the survival of MM cells.^49,65^ Cellular adhesion-mediated drug resistance (CAM-DR) is a well-documented phenomenon in MM, where ECM interactions protect myeloma cells from apoptosis, leading to lower sensitivity to inhibitors in 3D cultures compared to 2D cultures.^61,82-84^ Furthermore, studies have shown that hypoxia in 3D cultures can activate survival pathways such as HIF-1α, which promotes MM cell resistance to apoptosis.^65^ Additionally, as we aimed to study the effect of PI3K inhibitor on myeloma cells in different culture settings, the same concentrations of LY294002 was applied for both 2D and 3D treatment groups. Similarly, it has been reported that finding the effective drug concentration may be affected by the culture condition.^49,65^ These findings emphasize the importance of using 3D patient-derived culture systems to improve the predictive accuracy of preclinical drug screening. The discrepancies in cell death patterns observed in 2D versus 3D cultures may help explain why some MM treatments demonstrate efficacy in traditional *in vitro* models but fail in clinical trials.^54,85^ Understanding these differential responses can aid in designing more physiologically relevant *ex-vivo* models that better recapitulate the BM niche, ultimately enhancing the development of personalized therapeutic strategies for MM patients.^77,86,87^

 Moreover, in line with other studies, we also observed the inter-individual variability in response to the treatment. In a similar study, the effect of a broad-spectrum inhibitor of AKT (MK-2206) on myeloma cells was assessed in co-culture with patient BM-derived stromal cells in the 2D culture condition. The results showed that the inhibitor led to the patient-dependent decrease of the myeloma cell survival.^62^ The patient-dependent responses were also reported after treatment of MM BM-derived MNCs with GDC-0941, a class I PI3K inhibitor, in the 2D culture condition.^63^ Additionally, in another study, the MM-apoptotic induction effect of trabectedin was shown on primary cells from three different relapsed MM patients in patient and culture-dependent manners in 2D and 3D Matrigel-based spheroids.^88^ The inter-individual variability in response to treatments was also shown in a study that evaluated the drug sensitivity in 2D cultures of MM cells treated with selinexor, bortezomib and dexamethasone.^89^ Moreover, in another study, the treatment of MNCs derived from MM patients PB samples with thalidomide and IL-2 in 2D cultures led to different patient-based patterns of increased lysis of primary myeloma cells.^90^

 The varied cellular responses to MM inhibitors among different patients can be attributed to several interconnected factors, including genetic variability, microenvironment influences, tumor heterogeneity, clonal evolution, epigenetic modifications, drug resistance mechanisms and the presence of additional mutations. Genetic mutations in myeloma cells can confer resistance to specific inhibitors, leading to differences in cell treatment efficacy across patients.^91-93^ Additionally, as previously stated, TME plays a crucial role in modulating drug efficacy, as BM stromal cells and immune components, such as macrophages, secrete cytokines (e.g., IL-6, VEGF, TNF-α) that promote MM survival and alter drug responses; the composition of the TME differs among patients, affecting drug penetration and therapeutic outcomes.^94^ Macrophage polarization significantly influences drug responses, as M2 macrophages support MM survival while M1 macrophages promote apoptosis. Differences in macrophage polarization between patients may impact the effectiveness of PI3K inhibitors.^95,96^ Moreover, tumor heterogeneity, including the clonal evolution of tumor cells during disease progression, can give rise to subpopulations with distinct drug sensitivities, further contributing to variability in treatment responses.^97^ Epigenetic alterations, such as DNA methylation, histone modifications, and microRNA regulation, also contribute to MM heterogeneity and resistance mechanisms.^98,99^ Furthermore, the presence of additional mutations or alterations in signaling pathways can lead to variations in drug sensitivity among different patients.^100^ Studies have highlighted that mutations in drug target genes or dysregulation of drug efflux pumps (e.g., ABC transporters) can reduce intracellular drug accumulation, conferring resistance to MM inhibitors.^101^ Lastly, the pharmacokinetics and drug penetration in 3D cultures, which better mimic the *in vivo* microenvironment compared to traditional 2D cultures, may introduce additional complexities that influence treatment responses.^49,65^ Given these multiple layers of variability, understanding the molecular mechanisms underlying these differential responses is crucial for developing personalized therapeutic strategies and identifying predictive biomarkers to optimize MM treatment outcomes. Ongoing research efforts continue to explore these complexities to inform the design of more effective and targeted treatment approaches.^102-104^

 Macrophages are one of the most abundant and highly flexible cells in the TME, which influence the net response of microenvironment to therapeutics, especially immunotherapies.^14,18,66,105^ Our results indicated that the inhibitor treatment resulted into the dose-dependent increase in the ratio of M1/M2 macrophages in treated groups compared to the untreated control group, in both 2D and 3D culture conditions, which correlated the percentage of dead myeloma cells. PI3K signaling pathway is one of the most important pathways involved in the polarization of macrophages, which has been used for the reprogramming and cell fate control of macrophages.^33,106,107^ It has been shown that some cytokines, such as CCL-2, 3, and 14, with increased expression in myeloma cells, could mediate macrophage migration into the TME, and by the activation of PI3K and MAPK signaling pathways could facilitate the polarization of macrophages into the M2 phenotype, resulting into the myeloma cell proliferation and survival.^108^ Additionally, highly secreted factors in myeloma microenvironment, including TGF-β, IL-10, and BMP-7, could increase the macrophage polarization to the M2 phenotype through the activation of PI3K signaling pathway.^109-111^ Moreover, some studies have shown that suppressing the PI3K signaling pathway could increase the M1 macrophage phenotype.^31,33^ However, in a contrary study, LY294002 and IC87114 reduced the activation of inflammatory factors, including IL-6, MCP-1, TNF-α, and iNOS in activated macrophages in the carrageenan-induced paw oedema mouse model, by suppressing the AKT phosphorylation.^112^ Additionally, in another study, the LY294002 treatment reduced the phagocytic activity of macrophages in a dose-dependent manner.^113^ Moreover, the usage of this inhibitor led to the inhibition of antibody-dependent cellular cytotoxicity (ADCC) against B cell lymphoma in the 2D culture condition.^114^ Notably, these contradictory results are obtained from 2D cultures of cell lines or animal models, and therefore, the impacts of either cellular interactions, or genetic-associated differences and disease stages on the response to inhibitors are not considered. As mentioned previously, the recapitulation of TME using patient-derived cells in 3D culture conditions has gained significant attention and by considering the inter-individual differences, it is suggested to present the outcomes of these therapeutic approaches individually.^115,116^ In this regard, we presented the response of MM patient-derived primary cells to PI3K signaling pathway inhibitor on an individual patient basis, rather than averaging across multiple patients. This substantial variability in cellular responses aligns with previous studies highlighting the heterogeneous nature of MM and its patient-specific treatment responses.^104,117,118^ While our approach may provide valuable insights into patient-specific treatment responses, future studies incorporating larger patient cohorts will be essential to enable statistically robust analyses and further validate our findings.

 As one of the limitations of our study, we should note that the down-stream effects of the treatment with LY294002 were not evaluated, which requires future mechanistic experiments to confirm the PI3K signaling inhibition and to assess its probable cross-talks with other pathways. Moreover, histological assessments of generated 3D structures are highly recommended for future studies in order to follow the spatial localization of cells and to evaluate the underlying mechanisms of the increase in the M1/M2 ratio. Furthermore, mechanistic evaluations are required to confirm the correlation between the M1/M2 ratio and the MM cell fate, using more patient samples. Additionally, we utilized an L-glutamine-enriched medium to optimize U266 MM cell proliferation, as it is an essential nutrient that supports cell growth, survival, and metabolic activity.^119-122^ This approach was based on our previous research^47^ to maintain optimal culture conditions for MM cells in both 2D and 3D environments, ensuring that differences in cell viability were primarily due to the inhibitor rather than nutrient deprivation. However, we acknowledge that L-glutamine can influence PI3K/Akt signaling.^123^ Although this interfering effect might be mitigated by the presence of control groups, the possibility that L-glutamine modulates drug sensitivity cannot be entirely ruled out, potentially affecting cell survival and apoptosis mechanisms in MM cells.

## Conclusion

 This study evaluated the effects of LY294002 treatment on myeloma cell death and macrophage ratios under different conditions, including mono-culture of cell line versus primary culture of MNCs, and 2D versus 3D culture conditions. While the treatment of cell line showed the inhibitory effects of LY294002 on the MM survival, this homogenous pattern of cell death was not observed in patient-derived MNCs, indicating patient-dependent variability. Moreover, cell death responses differed between 2D and 3D cultures, emphasizing the influence of the microenvironment on treatment outcomes. These findings highlighted the necessity of better replicating the TME in experimental models and considering the inter-individual differences for more accurate therapeutic assessments.

## Competing Interests

 The authors have no competing interests to declare.

## Ethical Approval

 The study was performed based on the ethics protocols of Tarbiat Modares University, Faculty of Medical Sciences (code: IR.MODARES.REC.1399.228).

## Supplementary Files


Supplementary file 1 contains Figures S1-S6.

## References

[1] García-Ortiz A, Rodríguez-García Y, Encinas J, Maroto-Martín E, Castellano E, Teixidó J (2021). The role of tumor microenvironment in multiple myeloma development and progression. Cancers (Basel).

[2] Nwabuko OC (2023). Multiple myeloma: risk factors, pathogenesis and relationship with anti-myeloma therapies. J Explor Res Pharmacol.

[3] Huang J, Chan SC, Lok V, Zhang L, Lucero-Prisno DE 3rd, Xu W (2022). The epidemiological landscape of multiple myeloma: a global cancer registry estimate of disease burden, risk factors, and temporal trends. Lancet Haematol.

[4] Neumeister P, Schulz E, Pansy K, Szmyra M, Deutsch AJ (2022). Targeting the microenvironment for treating multiple myeloma. Int J Mol Sci.

[5] Desantis V, Savino FD, Scaringella A, Potenza MA, Nacci C, Frassanito MA (2022). The leading role of the immune microenvironment in multiple myeloma: a new target with a great prognostic and clinical value. J Clin Med.

[6] Forster S, Radpour R (2022). Molecular impact of the tumor microenvironment on multiple myeloma dissemination and extramedullary disease. Front Oncol.

[7] Schwestermann J, Besse A, Driessen C, Besse L (2022). Contribution of the tumor microenvironment to metabolic changes triggering resistance of multiple myeloma to proteasome inhibitors. Front Oncol.

[8] Hervás-Salcedo R, Martín-Antonio B (2022). A journey through the inter-cellular interactions in the bone marrow in multiple myeloma: implications for the next generation of treatments. Cancers (Basel).

[9] Schinke C, Weinhold N, Delgado-Calle J (2023). Editorial: the role of the bone marrow microenvironment in multiple myeloma evolution and therapy. Front Oncol.

[10] Hou J, Wei R, Qian J, Wang R, Fan Z, Gu C (2019). The impact of the bone marrow microenvironment on multiple myeloma (review). Oncol Rep.

[11] Nakamura K, Smyth MJ, Martinet L (2020). Cancer immunoediting and immune dysregulation in multiple myeloma. Blood.

[12] Swamydas M, Murphy EV, Ignatz-Hoover JJ, Malek E, Driscoll JJ (2022). Deciphering mechanisms of immune escape to inform immunotherapeutic strategies in multiple myeloma. J Hematol Oncol.

[13] Hu W (2023). Potential multiple myeloma therapeutic strategies through targeting macrophages and mesenchymal stromal cells. J Stud Res.

[14] Sun J, Park C, Guenthner N, Gurley S, Zhang L, Lubben B (2022). Tumor-associated macrophages in multiple myeloma: advances in biology and therapy. J Immunother Cancer.

[15] Sun M, Xiao Q, Wang X, Yang C, Chen C, Tian X (2021). Tumor-associated macrophages modulate angiogenesis and tumor growth in a xenograft mouse model of multiple myeloma. Leuk Res.

[16] Cencini E, Sicuranza A, Ciofini S, Fabbri A, Bocchia M, Gozzetti A (2023). Tumor-associated macrophages in multiple myeloma: key role in disease biology and potential therapeutic implications. Curr Oncol.

[17] Zheng Y, Cai Z, Wang S, Zhang X, Qian J, Hong S (2009). Macrophages are an abundant component of myeloma microenvironment and protect myeloma cells from chemotherapy drug-induced apoptosis. Blood.

[18] Haabeth OA, Hennig K, Fauskanger M, Løset G, Bogen B, Tveita A (2020). CD4 + T-cell killing of multiple myeloma cells is mediated by resident bone marrow macrophages. Blood Adv.

[19] Asimakopoulos F, Kim J, Denu RA, Hope C, Jensen JL, Ollar SJ (2013). Macrophages in multiple myeloma: emerging concepts and therapeutic implications. Leuk Lymphoma.

[20] Yang Y, Jia X, Qu M, Yang X, Fang Y, Ying X (2023). Exploring the potential of treating chronic liver disease targeting the PI3K/Akt pathway and polarization mechanism of macrophages. Heliyon.

[21] Kerneur C, Cano CE, Olive D (2022). Major pathways involved in macrophage polarization in cancer. Front Immunol.

[22] Tian T, Wang Z, Chen L, Xu W, Wu B (2023). Photobiomodulation activates undifferentiated macrophages and promotes M1/M2 macrophage polarization via PI3K/AKT/mTOR signaling pathway. Lasers Med Sci.

[23] Wang S, Liu G, Li Y, Pan Y (2022). Metabolic reprogramming induces macrophage polarization in the tumor microenvironment. Front Immunol.

[24] Vergadi E, Ieronymaki E, Lyroni K, Vaporidi K, Tsatsanis C (2017). Akt signaling pathway in macrophage activation and M1/M2 polarization. J Immunol.

[25] Hideshima T, Anderson KC (2021). Signaling pathway mediating myeloma cell growth and survival. Cancers (Basel).

[26] Mehdizadeh M, Farhadihosseinabadi B, Nikoonezhad M, Sankanian G, Soleimani M, Sayad A (2022). Phosphatidylinositol 3-kinase signaling inhibitors for treatment of multiple myeloma: from small molecules to microRNAs. J Oncol Pharm Pract.

[27] Safaroghli-Azar A, Bashash D, Kazemi A, Pourbagheri-Sigaroodi A, Momeny M (2019). Anticancer effect of pan-PI3K inhibitor on multiple myeloma cells: shedding new light on the mechanisms involved in BKM120 resistance. Eur J Pharmacol.

[28] Kikuchi H, Amofa E, McEnery M, Schey SA, Ramasamy K, Farzaneh F (2023). Inhibition of PI3K class IA kinases using GDC-0941 overcomes cytoprotection of multiple myeloma cells in the osteoclastic bone marrow microenvironment enhancing the efficacy of current clinical therapeutics. Cancers (Basel).

[29] Yang D, Yang L, Cai J, Li H, Xing Z, Hou Y (2022). Phosphoinositide 3-kinase/Akt and its related signaling pathways in the regulation of tumor-associated macrophages polarization. Mol Cell Biochem.

[30] Gunderson AJ, Kaneda MM, Tsujikawa T, Nguyen AV, Affara NI, Ruffell B (2016). Bruton tyrosine kinase-dependent immune cell cross-talk drives pancreas cancer. Cancer Discov.

[31] Li M, Li M, Yang Y, Liu Y, Xie H, Yu Q (2020). Remodeling tumor immune microenvironment via targeted blockade of PI3K-γ and CSF-1/CSF-1R pathways in tumor associated macrophages for pancreatic cancer therapy. J Control Release.

[32] Joshi S, Singh AR, Liu KX, Pham TV, Zulcic M, Skola D (2019). SF2523: dual PI3K/BRD4 inhibitor blocks tumor immunosuppression and promotes adaptive immune responses in cancer. Mol Cancer Ther.

[33] Joshi S, Liu KX, Zulcic M, Singh AR, Skola D, Glass CK (2020). Macrophage Syk-PI3Kγ inhibits antitumor immunity: SRX3207, a novel dual Syk-PI3K inhibitory chemotype relieves tumor immunosuppression. Mol Cancer Ther.

[34] Heinemann L, Möllers KM, Ahmed HM, Wei L, Sun K, Nimmagadda SC (2022). Inhibiting PI3K-AKT-mTOR signaling in multiple myeloma-associated mesenchymal stem cells impedes the proliferation of multiple myeloma cells. Front Oncol.

[35] Chen P, Wu S, Dong X, Zhou M, Xu P, Chen B (2022). Formosanin C induces autophagy-mediated apoptosis in multiple myeloma cells through the PI3K/AKT/mTOR signaling pathway. Hematology.

[36] Zheng Y, Yang J, Zhang L, Qian J, Matthews J, Wang M (2010). Novel PI3K inhibitor compound A induces myeloma cell apoptosis and shows synergistic cytotoxicity with dexamethasone in multiple myeloma. Blood.

[37] Ferrarini M, Mazzoleni G, Steimberg N, Belloni D, Ferrero E. Innovative models to assess multiple myeloma biology and the impact of drugs. In: Hajek R, ed. Multiple Myeloma-A Quick Reflection on the Fast Progress. IntechOpen; 2013. doi: 10.5772/54312.

[38] Abe M, Hiura K, Ozaki S, Kido S, Matsumoto T (2009). Vicious cycle between myeloma cell binding to bone marrow stromal cells via VLA-4-VCAM-1 adhesion and macrophage inflammatory protein-1alpha and MIP-1beta production. J Bone Miner Metab.

[39] Pampaloni F, Reynaud EG, Stelzer EH (2007). The third dimension bridges the gap between cell culture and live tissue. Nat Rev Mol Cell Biol.

[40] Thippabhotla S, Zhong C, He M (2019). 3D cell culture stimulates the secretion of in vivo like extracellular vesicles. Sci Rep.

[41] Zdzisińska B, Roliński J, Piersiak T, Kandefer-Szerszeń M (2009). A comparison of cytokine production in 2-dimensional and 3-dimensional cultures of bone marrow stromal cells of multiple myeloma patients in response to RPMI8226 myeloma cells. Folia HistochemCytobiol.

[42] Kirshner J, Thulien KJ, Martin LD, Debes Marun C, Reiman T, Belch AR (2008). A unique three-dimensional model for evaluating the impact of therapy on multiple myeloma. Blood.

[43] Wu D, Wang Z, Li J, Song Y, Perez ME, Wang Z (2022). A 3D-bioprinted multiple myeloma model. Adv Healthc Mater.

[44] Waldschmidt JM, Fruttiger SJ, Wider D, Jung J, Thomsen AR, Hartmann TN (2022). Ex vivo propagation in a novel 3D high-throughput co-culture system for multiple myeloma. J Cancer Res Clin Oncol.

[45] Kozalak G, Bütün İ, Toyran E, Koşar A (2023). Review on bortezomib resistance in multiple myeloma and potential role of emerging technologies. Pharmaceuticals (Basel).

[46] Hashemzadeh K, Jokar MH, Sedighi S, Moradzadeh M (2019). Therapeutic potency of PI3K pharmacological inhibitors of gastrointestinal cancer. Middle East J Dig Dis.

[47] Jomehpour M, Khosravi M, Janfada M, Abroun S, Vahdat S (2023). Establishment of a three-dimensional culture condition for the U266 cell line based on peripheral blood plasma-derived fibrin gels. Cell J.

[48] Pösel C, Möller K, Fröhlich W, Schulz I, Boltze J, Wagner DC (2012). Density gradient centrifugation compromises bone marrow mononuclear cell yield. PLoS One.

[49] Alhallak K, Jeske A, de la Puente P, Sun J, Fiala M, Azab F (2021). A pilot study of 3D tissue-engineered bone marrow culture as a tool to predict patient response to therapy in multiple myeloma. Sci Rep.

[50] Han K, Xu X, Chen G, Zeng Y, Zhu J, Du X (2014). Identification of a promising PI3K inhibitor for the treatment of multiple myeloma through the structural optimization. J Hematol Oncol.

[51] Liu R, Chen Y, Liu G, Li C, Song Y, Cao Z (2020). PI3K/AKT pathway as a key link modulates the multidrug resistance of cancers. Cell Death Dis.

[52] Wang L, Lin N, Li Y (2019). The PI3K/AKT signaling pathway regulates ABCG2 expression and confers resistance to chemotherapy in human multiple myeloma. Oncol Rep.

[53] Lu Q, Yang D, Li H, Niu T, Tong A (2024). Multiple myeloma: signaling pathways and targeted therapy. Mol Biomed.

[54] Papadimitriou K, Kostopoulos IV, Tsopanidou A, Orologas-Stavrou N, Kastritis E, Tsitsilonis O (2020). Ex vivo models simulating the bone marrow environment and predicting response to therapy in multiple myeloma. Cancers (Basel).

[55] Liu P, Xu B, Li J, Lu H (2008). LY294002 inhibits leukemia cell invasion and migration through early growth response gene 1 induction independent of phosphatidylinositol 3-kinase-Akt pathway. BiochemBiophys Res Commun.

[56] Kunnimalaiyaan M, Ndiaye M, Chen H (2006). Apoptosis-mediated medullary thyroid cancer growth suppression by the PI3K inhibitor LY294002. Surgery.

[57] Imai Y, Yoshimori M, Fukuda K, Yamagishi H, Ueda Y (2012). The PI3K/Akt inhibitor LY294002 reverses BCRP-mediated drug resistance without affecting BCRP translocation. Oncol Rep.

[58] Wu D, Tao J, Xu B, Qing W, Li P, Lu Q (2011). Phosphatidylinositol 3-kinase inhibitor LY294002 suppresses proliferation and sensitizes doxorubicin chemotherapy in bladder cancer cells. Urol Int.

[59] Xie X, Tang B, Zhou J, Gao Q, Zhang P (2013). Inhibition of the PI3K/Akt pathway increases the chemosensitivity of gastric cancer to vincristine. Oncol Rep.

[60] Wang YQ, Lin Y, Zhao JD, Yang YT (2017). [Inhibitory effect of LY294002 on proliferation of multiple myeloma cells and its mechanism]. Zhongguo Shi Yan Xue Ye Xue Za Zhi.

[61] Chen P, Wen X, Wang B, Hou D, Zou H, Yuan Q (2018). PI3K/Akt inhibitor LY294002 potentiates homoharringtonine antimyeloma activity in myeloma cells adhered to stromal cells and in SCID mouse xenograft. Ann Hematol.

[62] Ramakrishnan V, Kimlinger T, Haug J, Painuly U, Wellik L, Halling T (2012). Anti-myeloma activity of Akt inhibition is linked to the activation status of PI3K/Akt and MEK/ERK pathway. PLoS One.

[63] Munugalavadla V, Mariathasan S, Slaga D, Du C, Berry L, Del Rosario G (2014). The PI3K inhibitor GDC-0941 combines with existing clinical regimens for superior activity in multiple myeloma. Oncogene.

[64] Zhang J, Liu Z, Cao P, Wang H, Liu H, Hua L (2022). Tumor-associated macrophages regulate the function of cytotoxic T lymphocyte through PD-1/PD-L1 pathway in multiple myeloma. Cancer Med.

[65] de la Puente P, Muz B, Gilson RC, Azab F, Luderer M, King J (2015). 3D tissue-engineered bone marrow as a novel model to study pathophysiology and drug resistance in multiple myeloma. Biomaterials.

[66] Sun J, Muz B, Alhallak K, Markovic M, Gurley S, Wang Z (2020). Targeting CD47 as a novel immunotherapy for multiple myeloma. Cancers (Basel).

[67] Guzzeloni V, Veschini L, Pedica F, Ferrero E, Ferrarini M (2022). 3D models as a tool to assess the anti-tumor efficacy of therapeutic antibodies: advantages and limitations. Antibodies (Basel).

[68] Jeske A, Azab F, de la Puente P, Muz B, King J, Kohnen DR (2018). 3D-tissue engineered bone marrow (3DTEBM) culture retrospectively predicts treatment clinical outcomes of multiple myeloma patients. Blood.

[69] Calar K, Plesselova S, Bhattacharya S, Jorgensen M, de la Puente P (2020). Human plasma-derived 3D cultures model breast cancer treatment responses and predict clinically effective drug treatment concentrations. Cancers (Basel).

[70] Azab F, Vali S, Abraham J, Potter N, Muz B, de la Puente P (2014). PI3KCA plays a major role in multiple myeloma and its inhibition with BYL719 decreases proliferation, synergizes with other therapies and overcomes stroma-induced resistance. Br J Haematol.

[71] Sun CY, Hu Y, Huang J, Chu ZB, Zhang L, She XM (2010). Brain-derived neurotrophic factor induces proliferation, migration, and VEGF secretion in human multiple myeloma cells via activation of MEK-ERK and PI3K/AKT signaling. Tumour Biol.

[72] Liu Z, Zhang Y, Guo Y, Wang H, Fu R (2023). An overview of PIM kinase as a target in multiple myeloma. Cancer Med.

[73] Guenther A, Burger R, Klapper W, Tiemann M, Bakker F, Brocke-Heidrich K (2009). mTOR and PI3K inhibitors block myeloma cell growth in a synergistic manner. Blood.

[74] Huang YH, Almowaled M, Li J, Venner C, Sandhu I, Peters A (2021). Three-dimensional reconstructed bone marrow matrix culture improves the viability of primary myeloma cells in-vitro via a STAT3-dependent mechanism. Curr Issues Mol Biol.

[75] Stowers RS (2022). Advances in extracellular matrix-mimetic hydrogels to guide stem cell fate. Cells Tissues Organs.

[76] Jakubikova J, Cholujova D, Hideshima T, Gronesova P, Soltysova A, Harada T (2016). A novel 3D mesenchymal stem cell model of the multiple myeloma bone marrow niche: biologic and clinical applications. Oncotarget.

[77] Belloni D, Heltai S, Ponzoni M, Villa A, Vergani B, Pecciarini L (2018). Modeling multiple myeloma-bone marrow interactions and response to drugs in a 3D surrogate microenvironment. Haematologica.

[78] Peng X, Huang X, Lulu TB, Jia W, Zhang S, Cohen L (2024). A novel pan-PI3K inhibitor KTC1101 synergizes with anti-PD-1 therapy by targeting tumor suppression and immune activation. Mol Cancer.

[79] Zhong Z, Wang T, Zang R, Zang Y, Feng Y, Yan S (2024). Dual PI3K/mTOR inhibitor PF-04979064 regulates tumor growth in gastric cancer and enhances drug sensitivity of gastric cancer cells to 5-FU. Biomed Pharmacother.

[80] Cao Y, Luetkens T, Kobold S, Hildebrandt Y, Gordic M, Lajmi N (2010). The cytokine/chemokine pattern in the bone marrow environment of multiple myeloma patients. Exp Hematol.

[81] Schultz JC (1994). Leukaemic peripheral blood plasma and bone marrow plasma: comparison of influence on lymphocyte proliferation. Cell Prolif.

[82] Ilic J, Koelbl C, Simon F, Wußmann M, Ebert R, Trivanovic D (2024). Liquid overlay and collagen-based three-dimensional models for in vitro investigation of multiple myeloma. Tissue Eng Part C Methods.

[83] Bishop RT, Miller AK, Froid M, Nerlakanti N, Li T, Frieling JS (2024). The bone ecosystem facilitates multiple myeloma relapse and the evolution of heterogeneous drug resistant disease. Nat Commun.

[84] Bhowmick K, von Suskil M, Al-Odat OS, Elbezanti WO, Jonnalagadda SC, Budak-Alpdogan T (2024). Pathways to therapy resistance: the sheltering effect of the bone marrow microenvironment to multiple myeloma cells. Heliyon.

[85] Poornima K, Francis AP, Hoda M, Eladl MA, Subramanian S, Veeraraghavan VP (2022). Implications of three-dimensional cell culture in cancer therapeutic research. Front Oncol.

[86] Ballav S, Deshmukh AJ, Siddiqui S, Aich J, Basu S. Two-dimensional and three-dimensional cell culture and their applications. In: Zhan X, ed. Cell Culture-Advanced Technology and Applications in Medical and Life Sciences. IntechOpen; 2021. doi: 10.5772/intechopen.100382.

[87] Lourenço D, Lopes R, Pestana C, Queirós AC, João C, Carneiro EA (2022). Patient-derived multiple myeloma 3D models for personalized medicine-are we there yet?. Int J Mol Sci.

[88] Cucè M, Gallo Cantafio ME, Siciliano MA, Riillo C, Caracciolo D, Scionti F (2019). Trabectedin triggers direct and NK-mediated cytotoxicity in multiple myeloma. J Hematol Oncol.

[89] Giliberto M, Thimiri Govinda Raj DB, Cremaschi A, Skånland SS, Gade A, Tjønnfjord GE (2022). Ex vivo drug sensitivity screening in multiple myeloma identifies drug combinations that act synergistically. Mol Oncol.

[90] Davies FE, Raje N, Hideshima T, Lentzsch S, Young G, Tai YT (2001). Thalidomide and immunomodulatory derivatives augment natural killer cell cytotoxicity in multiple myeloma. Blood.

[91] Morgan GJ, Walker BA, Davies FE (2012). The genetic architecture of multiple myeloma. Nat Rev Cancer.

[92] Corre J, Munshi N, Avet-Loiseau H (2015). Genetics of multiple myeloma: another heterogeneity level?. Blood.

[93] Hu Y, Chen W, Wang J (2019). Progress in the identification of gene mutations involved in multiple myeloma. Onco Targets Ther.

[94] Anderson KC (2016). Progress and paradigms in multiple myeloma. Clin Cancer Res.

[95] Bui I, Bonavida B (2024). Polarization of M2 tumor-associated macrophages (TAMs) in cancer immunotherapy. Crit Rev Oncog.

[96] Chen P, Chen Y, Wang Y, Sharma A, Veronika LK, Weiher H, et al. Macrophage-Derived Pro-Inflammatory Cytokines Augment the Cytotoxicity of Cytokine-Induced Killer Cells by Strengthening the Nkg2d Pathway in Multiple Myeloma. 2024. Available from: https://ssrn.com/abstract=4907662. 10.1038/s41598-025-99289-x PMC12078699 40369131

[97] Keats JJ, Chesi M, Egan JB, Garbitt VM, Palmer SE, Braggio E (2012). Clonal competition with alternating dominance in multiple myeloma. Blood.

[98] Sadida HQ, Abdulla A, Al Marzooqi S, Hashem S, Macha MA, Akil AS (2024). Epigenetic modifications: key players in cancer heterogeneity and drug resistance. Transl Oncol.

[99] Mehra N, Sundaram S, Shah P, Rao A (2025). Epigenetic role of long non-coding RNAs in multiple myeloma. Curr Oncol Rep.

[100] Korde N, Roschewski M, Zingone A, Kwok M, Manasanch EE, Bhutani M (2015). Treatment with carfilzomib-lenalidomide-dexamethasone with lenalidomide extension in patients with smoldering or newly diagnosed multiple myeloma. JAMA Oncol.

[101] Kumar SK, Rajkumar V, Kyle RA, van Duin M, Sonneveld P, Mateos MV (2017). Multiple myeloma. Nat Rev Dis Primers.

[102] Morè S, Corvatta L, Manieri VM, Morsia E, Offidani M (2024). The challenging approach to multiple myeloma: from disease diagnosis and monitoring to complications management. Cancers (Basel).

[103] Engelhardt M, Kortüm KM, Goldschmidt H, Merz M (2024). Functional cure and long-term survival in multiple myeloma: how to challenge the previously impossible. Haematologica.

[104] Maura F, Rajanna AR, Ziccheddu B, Poos AM, Derkach A, Maclachlan K (2024). Genomic classification and individualized prognosis in multiple myeloma. J Clin Oncol.

[105] Opperman KS, Vandyke K, Psaltis PJ, Noll JE, Zannettino AC (2021). Macrophages in multiple myeloma: key roles and therapeutic strategies. Cancer Metastasis Rev.

[106] Khan SU, Khan MU, Azhar Ud Din M, Khan IM, Khan MI, Bungau S (2023). Reprogramming tumor-associated macrophages as a unique approach to target tumor immunotherapy. Front Immunol.

[107] Li X, Liu R, Su X, Pan Y, Han X, Shao C (2019). Harnessing tumor-associated macrophages as aids for cancer immunotherapy. Mol Cancer.

[108] Li Y, Zheng Y, Li T, Wang Q, Qian J, Lu Y (2015). Chemokines CCL2, 3, 14 stimulate macrophage bone marrow homing, proliferation, and polarization in multiple myeloma. Oncotarget.

[109] Gong D, Shi W, Yi SJ, Chen H, Groffen J, Heisterkamp N (2012). TGFβ signaling plays a critical role in promoting alternative macrophage activation. BMC Immunol.

[110] Rocher C, Singla DK (2013). SMAD-PI3K-Akt-mTOR pathway mediates BMP-7 polarization of monocytes into M2 macrophages. PLoS One.

[111] Zhang F, Wang H, Wang X, Jiang G, Liu H, Zhang G (2016). TGF-β induces M2-like macrophage polarization via SNAIL-mediated suppression of a pro-inflammatory phenotype. Oncotarget.

[112] Eräsalo H, Laavola M, Hämäläinen M, Leppänen T, Nieminen R, Moilanen E (2015). PI3K inhibitors LY294002 and IC87114 reduce inflammation in carrageenan-induced paw oedema and down-regulate inflammatory gene expression in activated macrophages. Basic Clin PharmacolToxicol.

[113] Cox D, Tseng CC, Bjekic G, Greenberg S (1999). A requirement for phosphatidylinositol 3-kinase in pseudopod extension. J Biol Chem.

[114] Joshi T, Ganesan LP, Cheney C, Ostrowski MC, Muthusamy N, Byrd JC (2009). The PtdIns 3-kinase/Akt pathway regulates macrophage-mediated ADCC against B cell lymphoma. PLoS One.

[115] Reiman LT, Walker ZJ, Babcock LR, Forsberg PA, Mark TM, Sherbenou DW (2021). A case for improving frail patient outcomes in multiple myeloma with phenotype-driven personalized medicine. Aging Cancer.

[116] Pawlyn C, Davies FE (2019). Toward personalized treatment in multiple myeloma based on molecular characteristics. Blood.

[117] Yahng SA, Kim HJ, Lee SB, Yoo SH, Yoon JH, Lee JH (2024). Development of a personalized microfluidic platform for improving treatment efficiency in multiple myeloma. Blood.

[118] Lipof JJ, Abdallah N, Lipe B (2024). Personalized treatment of multiple myeloma in frail patients. Curr Oncol Rep.

[119] Munasinghe M, Athapaththu AM, Gunathilaka HN, Abeyewickreme W. Optimization of the Cell Culture Media to Obtain the Most Effective Nutrient Concentrations in the Medium for the Growth and Maintenance of the Myeloma Cells. Sri Lanka: University of Peradeniya; 2015.

[120] Zhong Y, Xu S, Liu Z (2022). The potential of glutamine metabolism-related long non-coding RNAs (lncRNAs) as prognostic biomarkers in multiple myeloma patients. Ann Transl Med.

[121] Chiu M, Toscani D, Marchica V, Taurino G, Costa F, Bianchi MG (2020). Myeloma cells deplete bone marrow glutamine and inhibit osteoblast differentiation limiting asparagine availability. Cancers (Basel).

[122] Bolzoni M, Chiu M, Accardi F, Vescovini R, Airoldi I, Storti P (2016). Dependence on glutamine uptake and glutamine addiction characterize myeloma cells: a new attractive target. Blood.

[123] Chen L, Cui H (2015). Targeting glutamine induces apoptosis: a cancer therapy approach. Int J Mol Sci.

