# Supplementary material for: PI3K Signaling Pathway Inhibitor Affects Myeloma Cells in a Culture-Dependent Manner
Source: Adv Pharm Bull. 2025 Apr 24;15(2):440–52. doi: 10.34172/apb.025.42774 (PMC12413969; doi:10.34172/apb.025.42774)
Supplement: Supplementary file 1 — contains Figures S1-S6. [file apb-15-440-s001.pdf]

Adv Pharm Bull. 2025;15(2):440-S1
doi: 10.34172/apb.025.42774
https://apb.tbzmed.ac.ir

Supplementary file 1

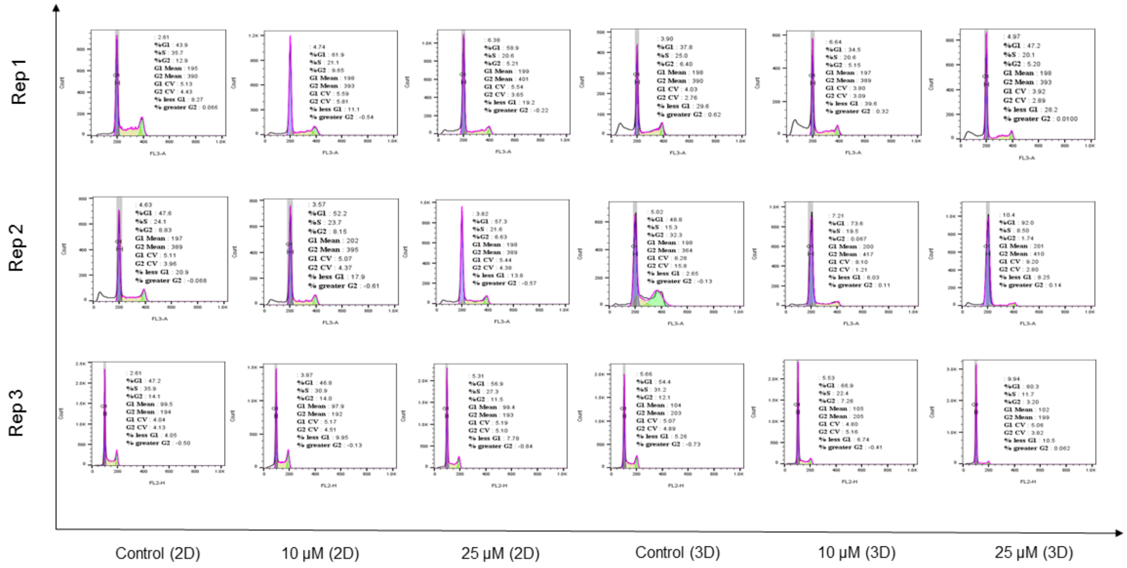

Figure S1. The cell cycle analysis of U266 cells after two days treatment with LY294002.

Patient 1 (CD138$^+$PI$^+$)

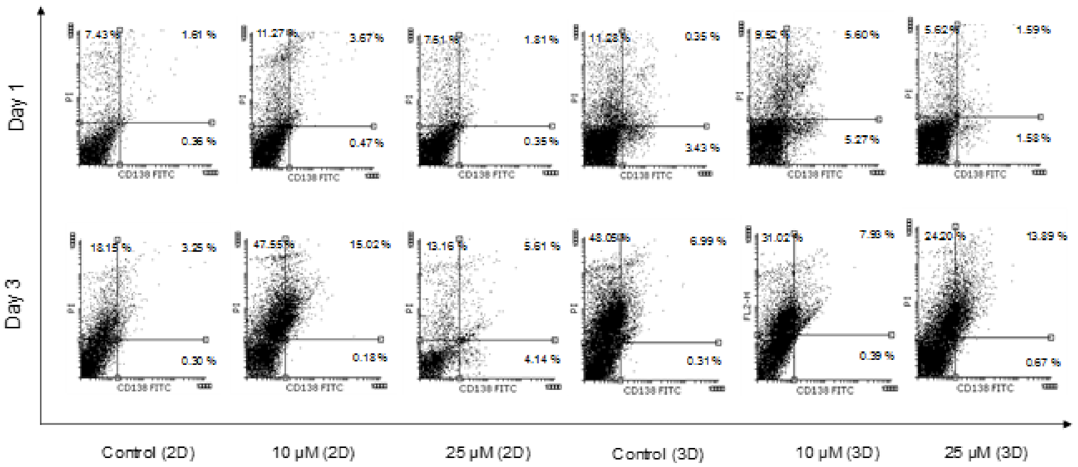

Patient 2 (CD138$^+$PI$^+$)

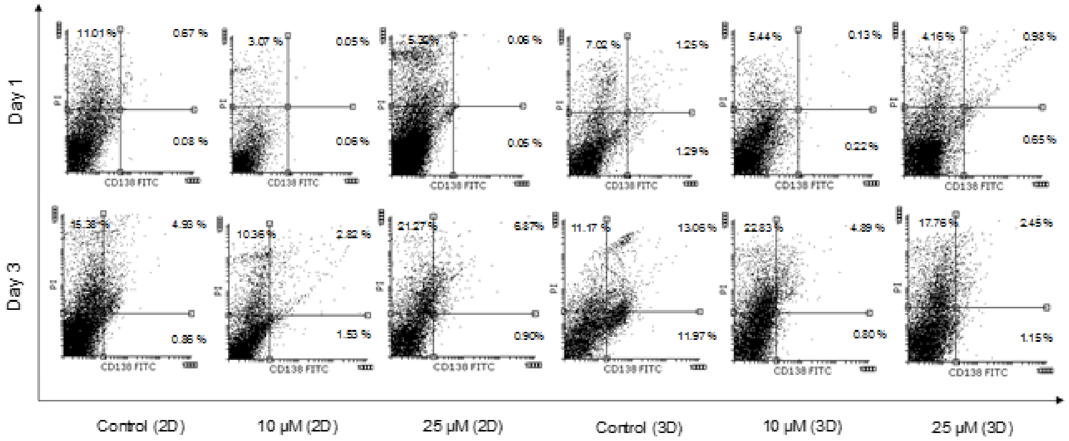

Patient 3 (CD138$^+$PI$^+$)

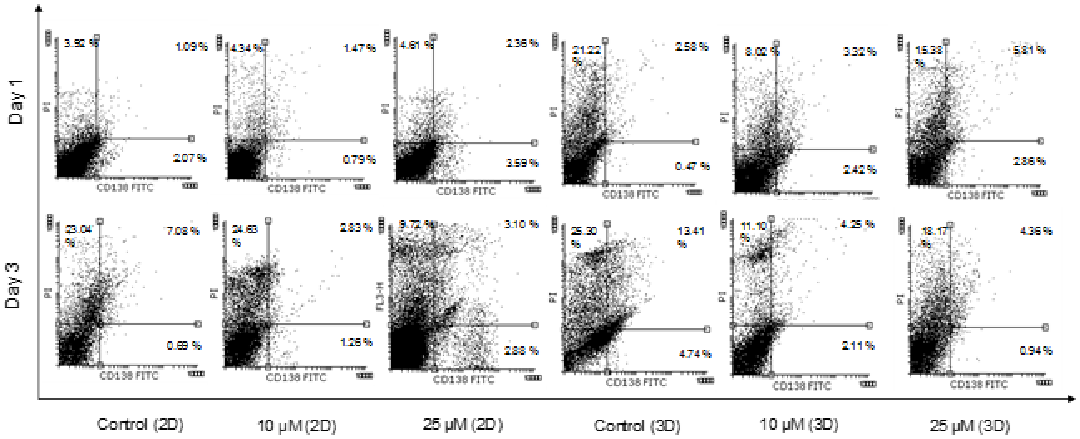

Figure S2. Flow cytometry diagrams of CD138⁺/ PI⁺ primary myeloma cells.

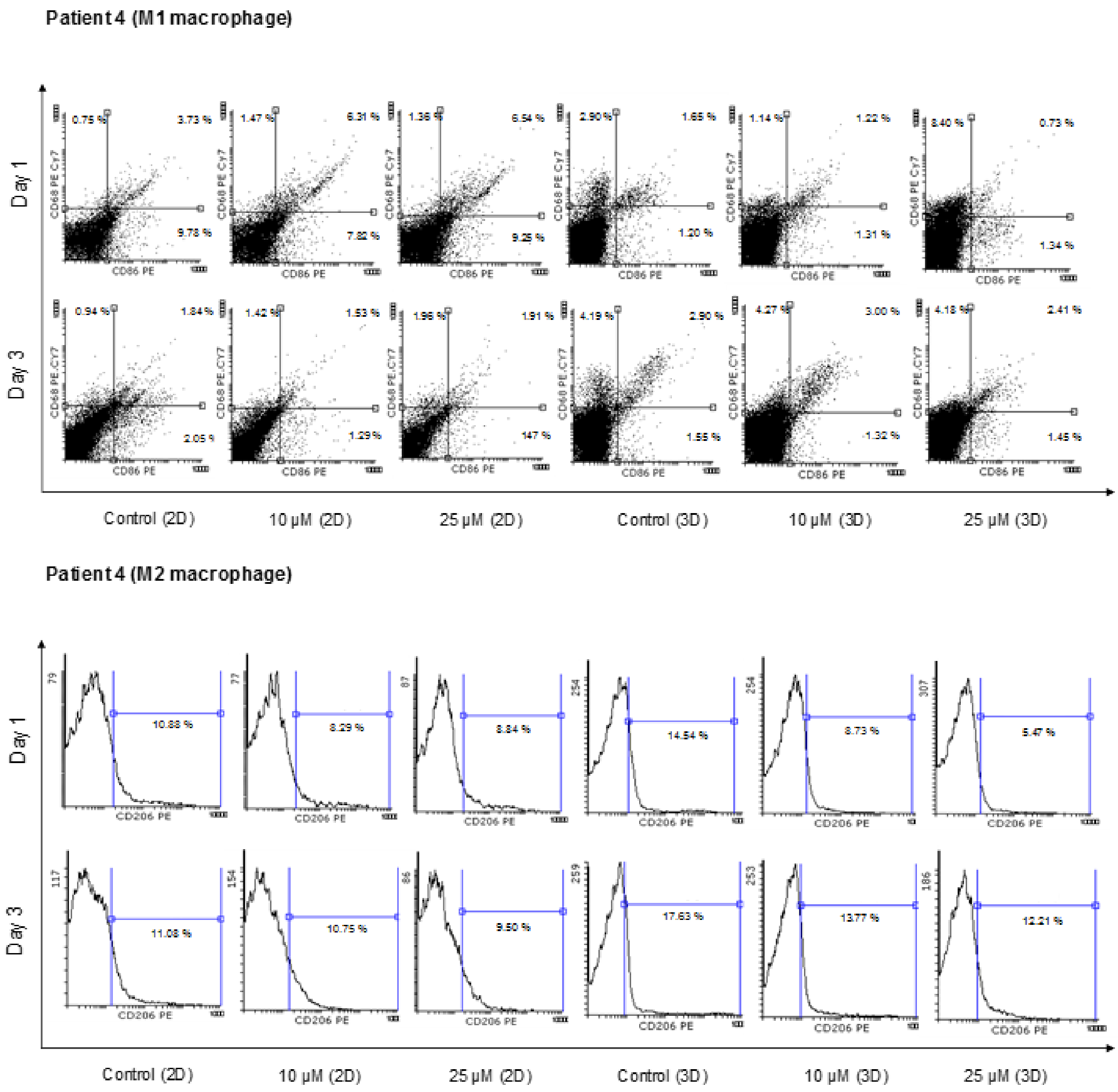

Figure S3. Macrophage immunophenotyping of patient 4-derived MNCs after treatments with LY294002.

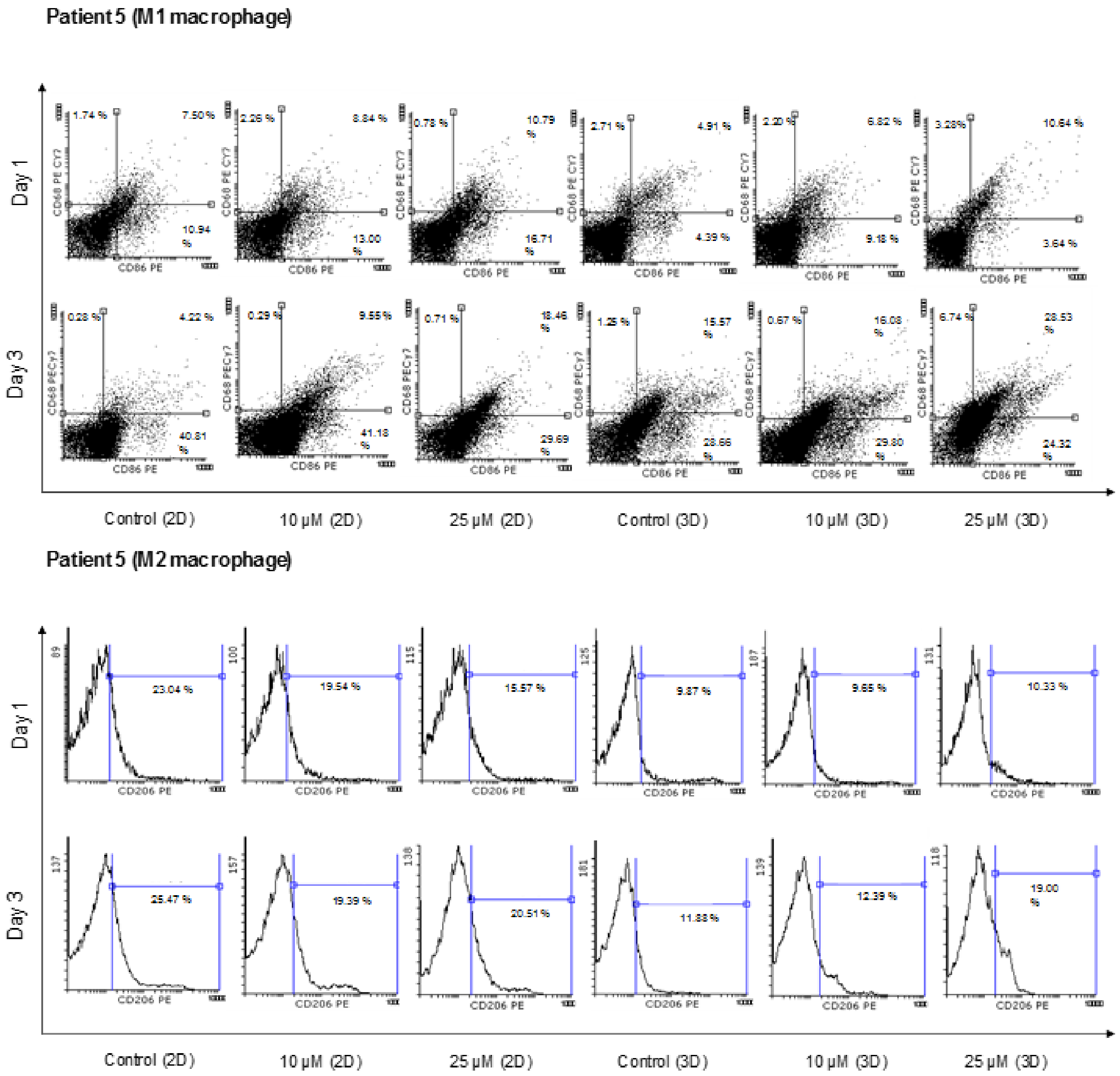

Figure S4. Macrophage immunophenotyping of patient 5-derived MNCs after treatments with LY294002.

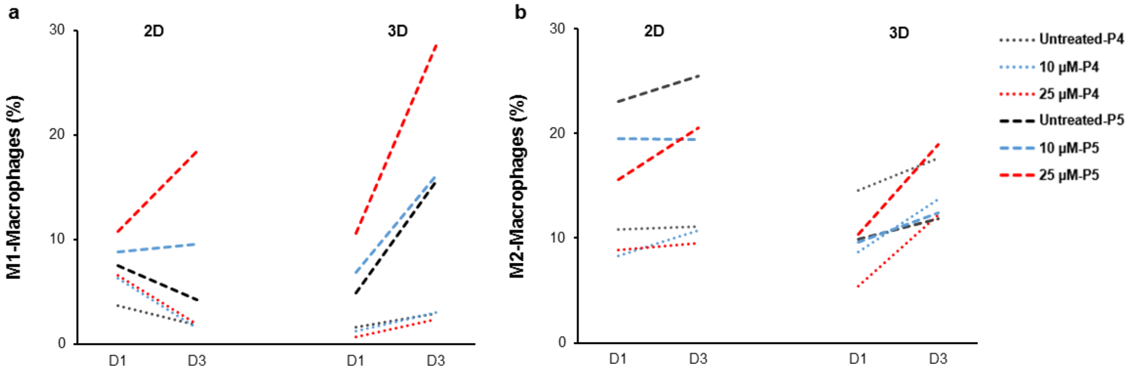

Figure S5. Macrophage immunophenotyping graphs of patient-derived MNCs after treatments with LY294002.

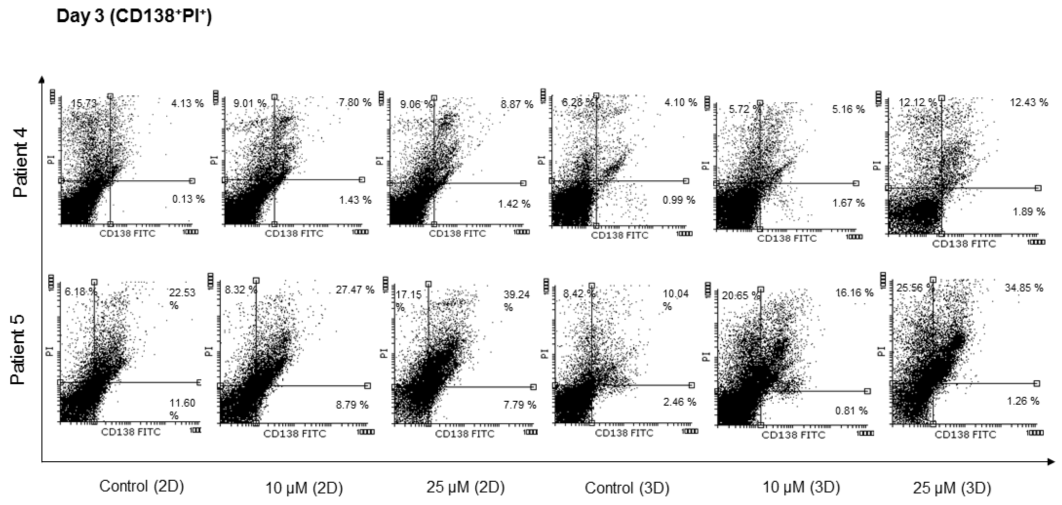

Figure S6. Flow cytometry diagrams of CD138$^+$/ PI$^+$ primary MNCs derived from patients 4 and 5 after treatments with LY294002.
